# Computed Tomography Imaging Findings of Acute Aortic Pathologies

**DOI:** 10.7759/cureus.5534

**Published:** 2019-08-30

**Authors:** Elif S Duran, Farhan Ahmad, Mohamed Elshikh, Irfan Masood, Cihan Duran

**Affiliations:** 1 Radiology, University of Texas Health Rio Grande Valley School of Medicine, Edinburg, USA; 2 Radiology, University of Texas Medical Branch at Galveston, Galveston, USA

**Keywords:** acute aortic syndrome, aortic dissection, intramural hematoma, penetrating atherosclerotic ulcer, multidetector-row computed tomography

## Abstract

Acute aortic syndromes (AAS) encompass a spectrum of life-threatening conditions characterized by acute aortic pain. AAS include acute aortic dissection, intramural hematoma, penetrating atherosclerotic ulcer, and aneurysm rupture. The prognosis of AAS is clearly related to prompt diagnosis and appropriate management. The different types of AAS cannot be reliably differentiated solely based on clinical presentation since the clinical features are indistinguishable. Multidetector-row computed tomography (MDCT) with electrocardiographic gating (ECG-gated MDCT) has been used in the acute emergency setting as a powerful clinical tool, which enables rapid and specific diagnosis of aortic pathologies. ECG-gated MDCT significantly reduces motion artifact and avoids potential pitfalls in the diagnosis of AAS. The aim of this review is to evaluate the role of MDCT imaging in the assessment of AAS and to discuss the differentiation of this spectrum of aortic diseases with reference to the key imaging findings.

## Introduction and background

Acute aortic syndromes describe the acute presentation of patients with characteristic aortic pain caused by one of several life-threatening conditions of aortic diseases. AAS is an umbrella term that traditionally includes four aortic diseases: aortic dissection (AD), intramural hematoma (IMH), penetrating atherosclerotic ulcer (PAU), and rupture of an aortic aneurysm, often due to trauma [1-3]. The incidence of AAS is 2.6-3.5 cases/100,000/year; two-thirds are male, with an average age of 63 years [4]. Predisposing factors, such as hypertension, Marfan's syndrome, atherosclerosis, cardiac surgery, inflammatory changes, and thrombi, are important in the process of identifying patients at risk both in adult and juvenile populations [5-7]. The clinical presentation of AAS is a real-time emergency, often presenting with acute chest pain, varyingly described as severe, tearing, or migratory [2,8]. Correlation of the clinical history, cardiac enzymes, and electrocardiography (ECG) can help differentiate AAS from acute coronary syndrome, however, it is essential to remember that acute coronary syndrome may occur as a result of AAS [3,8].

Multidetector-row computed tomography (MDCT) with electrocardiographic gating (ECG-gated MDCT), trans-esophageal echocardiography (TEE), and magnetic resonance imaging (MRI) are valuable tests in the diagnosis of AAS. TEE and MRI could be considered complementary modalities in defining particular diagnostic aspects, such as the functional evaluation of an aortic valve, heart function, floating thrombus, or in the case of a contraindication of iodinated contrast agent. The major advantage of echocardiography is the assessment of valve lesions, specifically aortic regurgitation in AAS [9]. The role of chest radiography for diagnosis or surveillance in this setting is very limited [10]. Conventional catheter angiography may be preferred as a means for treating complications of the disease. The main objectives of imaging are to confirm the diagnosis of an aortic wall lesion and to ascertain the site, extension, and complications of the disease. MDCT angiography is the modality of choice to evaluate patients with suspected AAS, as it has rapid acquisition and high diagnostic accuracy in detecting of the AAS [11-12].

In this review, our aim is to illustrate the MDCT findings of the most frequently encountered non-traumatic aortic emergencies. The imaging technique and typical imaging findings of the AAS will be discussed in this review.

## Review

MDCT angiography imaging technique in acute aortic syndrome

The MDCT imaging protocol depends on the technical characteristics of tomography available such as the number of detectors (16, 64, 128, 256, 320), rotation tube speed, and table feeding. Typically, axial reconstruction thickness should be between 1 mm and 3 mm, using 16 x 1.25 mm on 16-row scanners, 64 x 0.5 mm on 64-row scanners and 128 x 0.6 mm on the new 128-row scanners. Sagittal, coronal, and multiplanar reconstructions (MPRs) should be generated on three-dimensional (3D) workstations [11-13]. The protocol should be optimized to reduce examination times, to improve spatial resolution, and to apply ideal total contrast material volume and exposure dose.

Non-contrast images provide important information concerning the presence of calcification and intramural hematoma, size of the aorta, general status of the lung parenchyma, mediastinum, and heart size, presence of pleural effusion, abdominal organs, bowel, intra, and retroperitoneal space, and fasciae fluid collections [14-15].

The contrast agent administration protocol is based on the patient’s weight and potential abnormalities in kidney function. After a variable time of 10 to 30 seconds after contrast media injection, intravascular contrast enhancement increases and lumen opacification appears linearly correlated with dilution effect; this is influenced by several parameters, such as contrast media concentration, flow rate and pressure of infusion, cardiac output, scan parameters, and the presence of saline flush following the injection of contrast media when double injectors are used. Bolus timing is crucial in MDCT angiography; therefore, especially in critical patients, the automatic detection of bolus such as bolus tracking may be utilized. The protocol of contrast agent delivery depends on the characteristics of computed tomography available. Generally, non-ionic iodinated contrast agent at a high concentration (≥350 mg/ml) at a dosage max of 0.1- 0.2 ml/kg body weight with moderate-high flow rate (4-4.5 ml/s) with a bolus (30-50 ml) saline solution at the same flow rate following contrast injection using a double pump injector, could be considered the simplest and most efficient protocol, especially in emergency cases. The contrast agent dosage should be calculated in relation to the scan duration in order to avoid the scan time to exceed the infusion time delivery [16-18].

ECG-gated MDCT of the thoracic aorta significantly reduces the motion artifacts when compared with non-ECG-gated studies [19-21]. ECG gating of the aorta and coronary arteries can be performed either prospectively or retrospectively. During prospective ECG gating, the image is obtained usually during late diastole. However, this method is particularly susceptible to artifacts due to rapid changes in heart rate. The retrospective ECG-gating method reveals data continuously throughout the cardiac cycle. Images can also be viewed at any point along the R-R interval, thus allowing the selection of the phase with the least motion artifact for reconstruction [22]. However, the retrospective method causes higher radiation exposure than prospective ECG gating due to continuous versus intermittent X-ray exposure [23].

Aortic dissection

Aortic dissection is characterized by the separation of the aortic intima from the media, caused by the shearing forces of blood under high pressure, with variable longitudinal and circumferential extension, resulting in the formation of a double-channel aorta [24]. High blood pressure with the concomitant degenerative changes in the aortic media is the most common trigger for aortic dissection. Marfan syndrome, Turner syndrome, other connective tissue diseases, congenital aortic valvular defects, aortic coarctation, aortic aneurysm, aortitis, and pregnancy are among the most common causes of separation of the aortic intima and media. Since the right lateral wall of the ascending aorta and the proximal segment of the descending thoracic aorta have maximum hydraulic stress, intimal tears, leading to aortic dissections, often occur in these sites of the aorta [1,5,25].

The clinical presentation of aortic dissection can be very misleading and findings on physical examination may be non-specific. Patients may present with a classic history of acute-onset central chest pain that radiates to the back. Syncope can result from acute dissections and occurs in 9% of cases; syncope may be caused by hypotension secondary to cardiac tamponade, aortic rupture, cerebral vessel obstruction, or the activation of cerebral baroreceptors [26-27].

**Figure 1:**
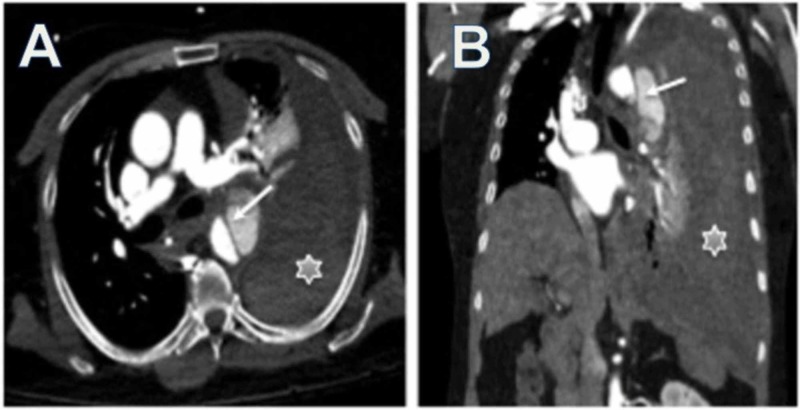
Type B Aortic Dissection Axial oblique (A) and coronal oblique (B) reformats and 3D volume rendering image of thoracic CT angiography images demonstrate Stanford type B thoracic aortic dissection (arrow) and rupture, with left hemothorax, pleural effusion, and near complete atelectasis of the left lung (star).

The intimal tear causes blood to enter the media from the vessel lumen. The blood-filled space within the medial layer creates a false lumen. This results in two lumina: a true and a false lumen with the false lumen having pressures greater than or equal to those in the true lumen [28]. Because of pressure differences, the false lumen may compress or obstruct the true lumen. Thus, the dissection can move in either an antegrade or a retrograde direction. The dissections may remain patent as a false lumen, thrombose, recommunicate with the true lumen through fenestrations, or rupture (Figure 1) into potential spaces such as the pericardial, pleural, or peritoneal cavities [29].

**Figure 2:**
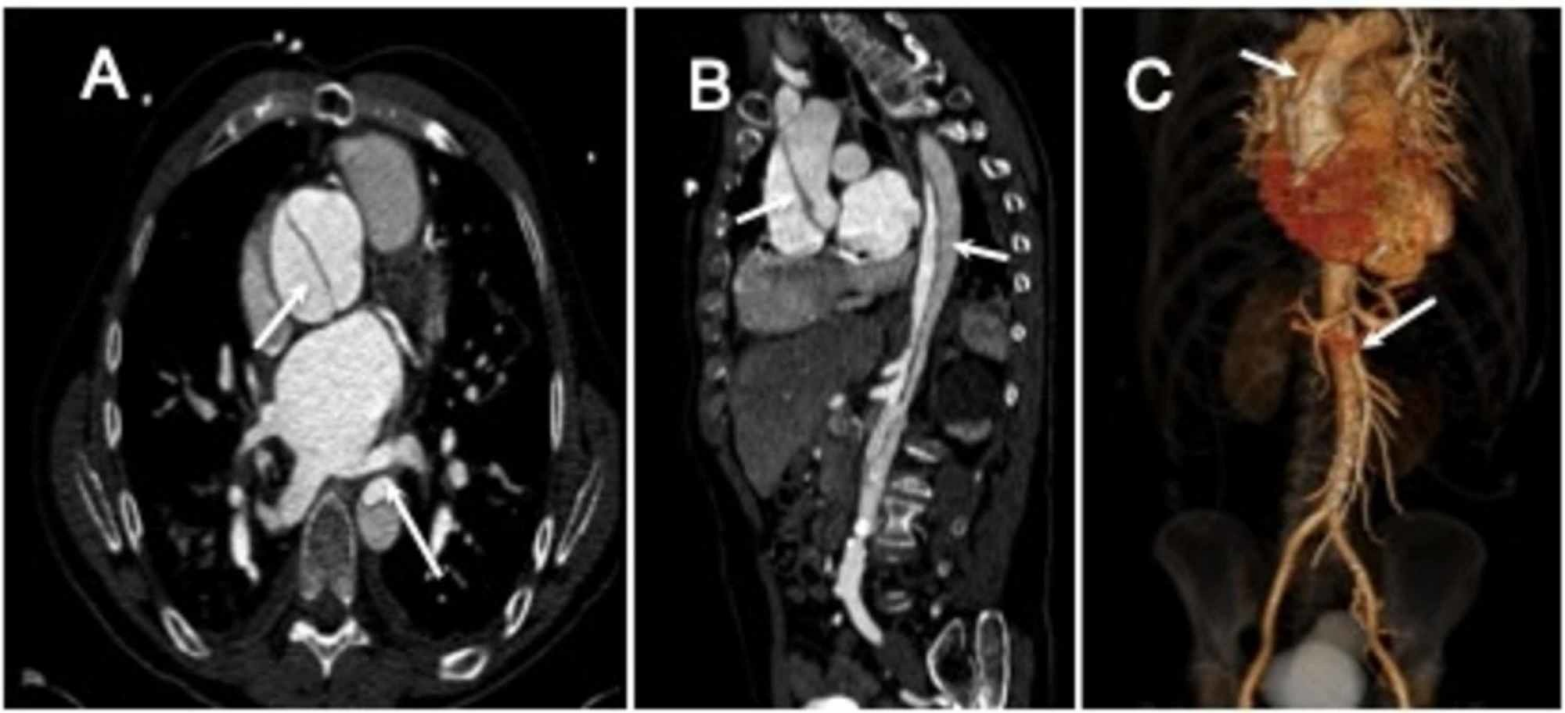
Type A Aortic Dissection A. Axial contrast-enhanced CT image demonstrating an intimal flap (arrows) consistent with a Stanford Type A dissection with extension into the distal thoracic aorta. B. 3D volume rendering image of the thoracic aorta visualization of the type A dissection (arrows). C. Sagittal reformat image shows the extension of the intimal flap into the abdominal aorta (arrows). The false lumen hypoattenuating as compared to the true lumen.

The classification of aortic dissection is based on the location and extension of the dissection, and the DeBakey and Stanford classification systems are most commonly utilized [30-31]. In general, the Stanford classification is preferred due to its capacity to propose immediate clinical management: surgical (type A) versus medical (type B) [1,5]. Stanford type A dissection includes the ascending aorta and can extend into the descending aorta (Figure 2). Stanford type B dissection includes the descending aorta beyond the origin of the left subclavian artery [30].

A widened mediastinum is the most common imaging finding on the radiography. In a study published in JAMA, a widened mediastinum was noted in 61.1% of aortic dissection cases, displacement of calcification of the aorta was reported in 14.1% of cases, with an abnormal cardiac contour being noted in 25.8% [32]. TEE has a reported sensitivity of 59%-83% and a specificity of 63%-93% for the diagnosis of aortic dissection. The sensitivity of transthoracic echocardiography is between 78% and 100% for the diagnosis of a type A dissection, but it is only 31%-55% for dissections involving the descending aorta [30,33].

In practice, ECG-gated MDCT should be preferred since it allows more precise delineation of the proximal extent of the intimal flap in relation to the aortic valve and coronary arteries and, more importantly, helps avoid the overdiagnosis of aortic dissections caused by the misinterpretation of a motion artifact as an intimal flap. Non-contrast MDCT images help obtain the extent of inward displacement of intimal calcification. The two most useful indicators of the false lumen are the beak sign and the cobweb sign [1,5,27]. The differences between true and false lumen MDCT imaging findings are summarized in Table 1.

**Table 1:** MDCT findings to differentiate true and false lumen MDCT: multidetector-row computed tomography

True Lumen	False Lumen
Smaller than a false lumen	Larger than a true lumen
Directly communicates with the aorta	Not connected to the unaffected aorta
Intima displaced inwards	Beak sign: Acute angle in the corner of the false lumen with true lumen
Calcification along the intimal flap	Cobwebs sign: Band of connective tissue crossing the false lumen
Calcification along the intimal flap	The surface of the intimal flap is convex
More enhanced than false lumen during the peak of aortic enhancement	Hypodense compared to the true lumen during the peak of aortic enhancement due to the presence of slow flow
Wrapped around the false lumen	Wrapped around the true lumen

Contrast-enhanced magnetic resonance angiography is more available for the investigation of aortic dissection in medically stable patients or those with chronic dissections. It has several advantages over MDCT angiography, including a lack of non-ionizing radiation, multiplanar evaluation, and greater vessel coverage at high resolution. Three-dimensional magnetic resonance angiography can reveal a complete and dynamic display of aortic dissection and display the true and false lumina [34-35].

Recently, triple rule-out MDCT protocol is used to assess the aorta, coronary arteries, and pulmonary arteries during a single scan with the use of several optimally timed boluses of contrast material and ECG gating in patients who are at low risk for an acute coronary syndrome. The main goal is to minimize the contrast material dose and radiation exposure while achieving optimal image quality, providing coronary artery image quality equivalent to that of dedicated coronary MDCT angiography, pulmonary artery image quality equivalent to that of dedicated pulmonary MDCT arteriography, and high-quality images of the thoracic aorta without pulsation artifact. In addition, the presence of acute coronary syndrome and aortic dissection can be evaluated via a triple rule-out technique [34]. This technique can evaluate and rule out pulmonary emboli, aortic coronary syndrome, and AAS all through the same imaging study.

Intramural hematoma

Intramural hematoma (IMH) is a variant of dissection and is characterized by the presence of hemorrhage into the aortic media from the vasa vasorum. Tears seen in classic aortic dissection are absent. IMHs are thought to comprise 10%-30% of all AAS [36-37]. IMH may originate spontaneously as a consequence of a penetrating ulcer or after thoracic trauma. It may be a precursor of aortic dissection, and many investigators have suggested that IMH is synonymous with a thrombosed type or noncommunicating aortic dissection [9]. Between the 50% and 85% of IMH occur in the descending aorta and are typically associated with hypertension. IMH is the cause of 5%-20% of acute aortic dissections. The clinical findings of IMH are similar to those of other acute aortic syndromes and patients predominantly present with acute chest pain [5,28].

**Figure 3:**
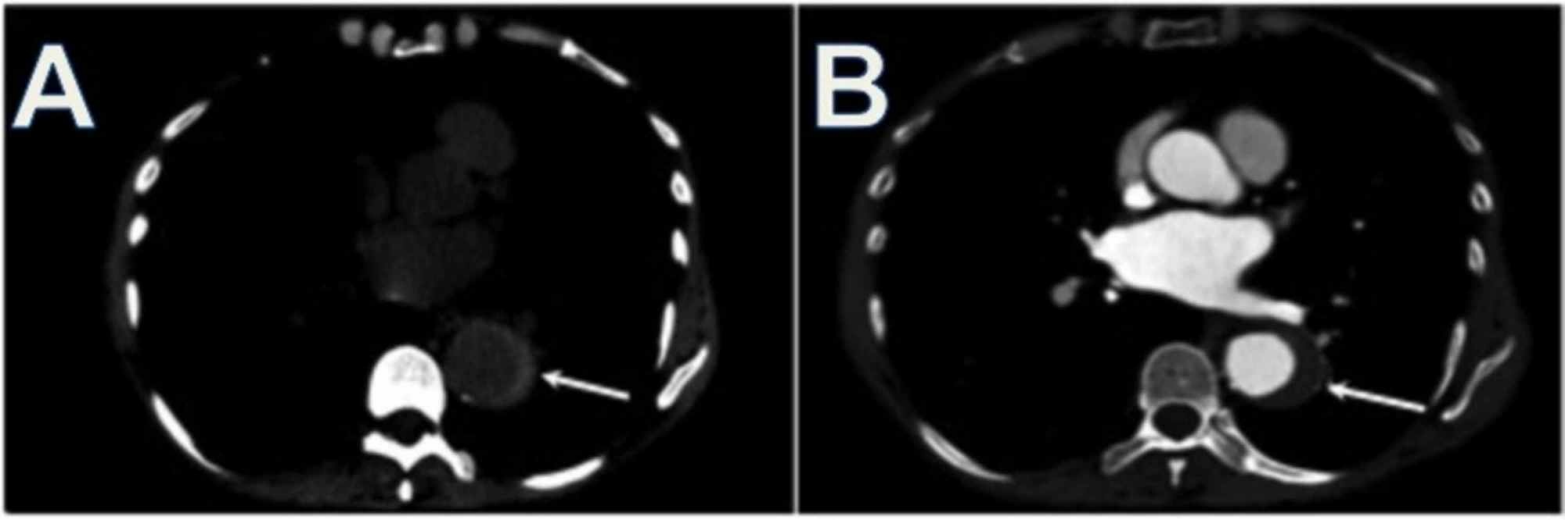
Intramural Hematoma A. Intramural hematomas is visualized as a crescent-shaped or ring like hyperdensity on non-contrast enhanced CT imaging. B. Contrast-enhanced CT demonstrates crescent-shaped hypoattenuation, not to be confused with atherosclerotic thrombus.

**Figure 4:**
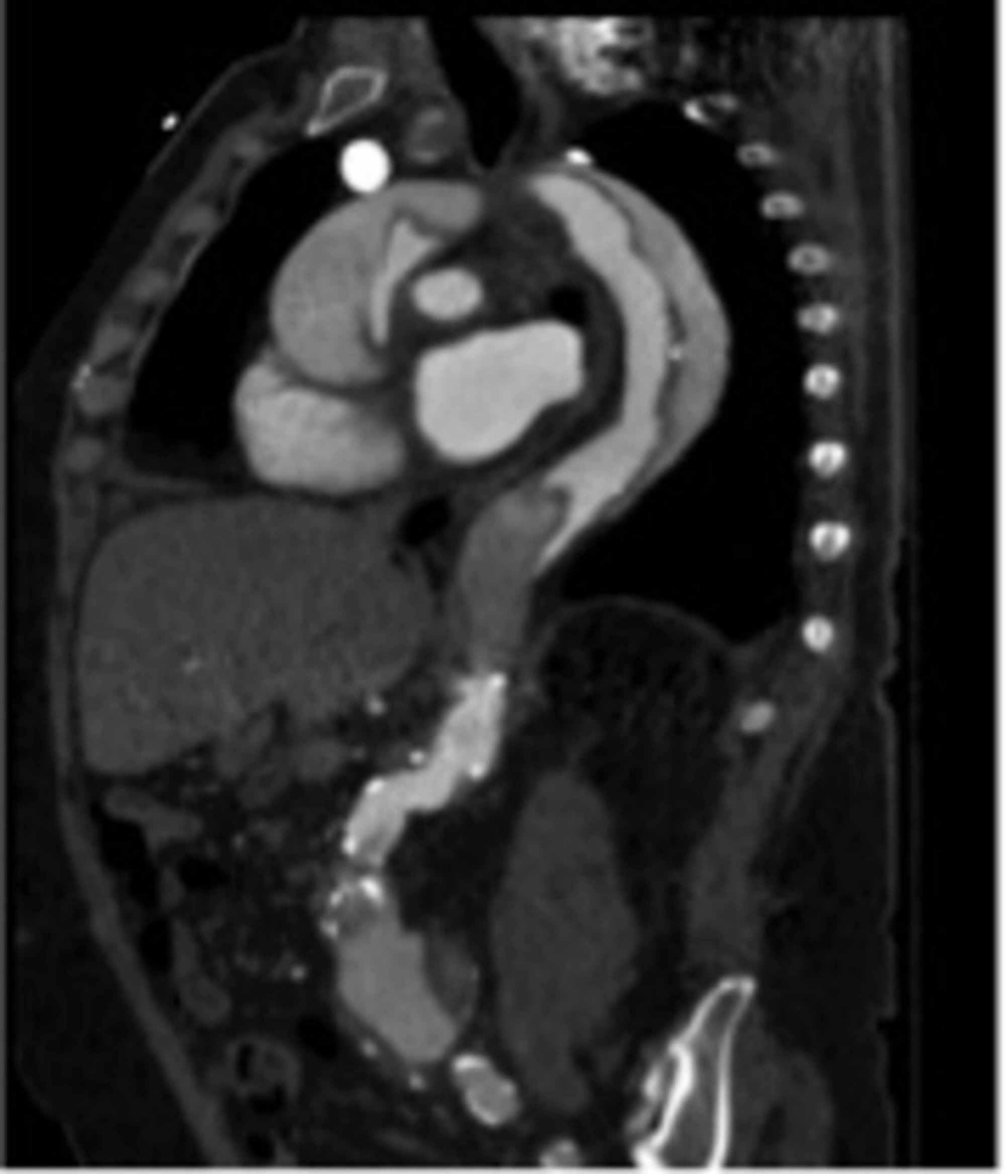
Aortic Dissection Spiraling Spiral course of the dissection flap of the aorta is seen in the sagittal reformat image.

The hyperdense crescent-shaped or ring-like thickening of the aortic wall is often detected on the non-contrast MDCT images, and precontrast imaging is essential in this protocol (Figure 3). The absence of an obvious communication between the true and false lumen explains the absence of flow and the lack of enhancement with contrast administration on MDCT or MRI [1,11]. On contrast-enhanced MDCT series, IMH may be easily confused with atherosclerotic thrombus, as the mildly increased attenuation of IMH compared to thrombus may be overlooked by the window level settings used to look at contrast-enhanced images. On the contrary to aortic dissection, the configuration of the IMH usually does not spiral around the aortic lumen (Figure 4). Additionally, IMH cannot be easily differentiated on the contrast-enhanced images. However, the thrombosed false lumen in classic aortic dissection has a pattern that spirals longitudinally around the aorta while an IMH pattern generally maintains a circumferential and eccentric relationship with the aortic wall. The advanced spatial resolution of MDCT allows us to visualize these features and differentiate the two pathologies [38-39].

Progression to aortic dissection occurs in 28%-47% of patients with IMH [40]. Similar to Stanford`s classification in the aortic dissection, surgery is suggested in patients with type A IMH and initial medical therapy is suggested in patients with type B IMH [41].

Penetrating atherosclerotic ulcer

An atherosclerotic plaque erodes the internal elastic lamina into the media of the aortic wall in patients with a penetrating atherosclerotic ulcer (PAU). These ulcers may be complicated by true aneurysm formation, erosion through the media to form a pseudoaneurysm or dissection [1,42]. PAU generally occurs in an elderly individual with multiple risk factors for atherosclerosis and the associated comorbidities of atherosclerotic disease such as coronary artery disease and peripheral arterial disease. The clinical findings of PAU may usually be the same as those of aortic dissection. In the absence of atherosclerosis, it can also occur in young patients with connective tissue disorder or after the rupture of a mycotic plaque [40,43]. Since the atheromatous plaque can rupture and precipitate intramural hemorrhage, early diagnosis of the PAU is essential. Most cases with PAU (approximately >90%) occur in the aortic arch or descending aorta; atherosclerotic plaques are rarely located in the ascending aorta [44].

**Figure 5:**
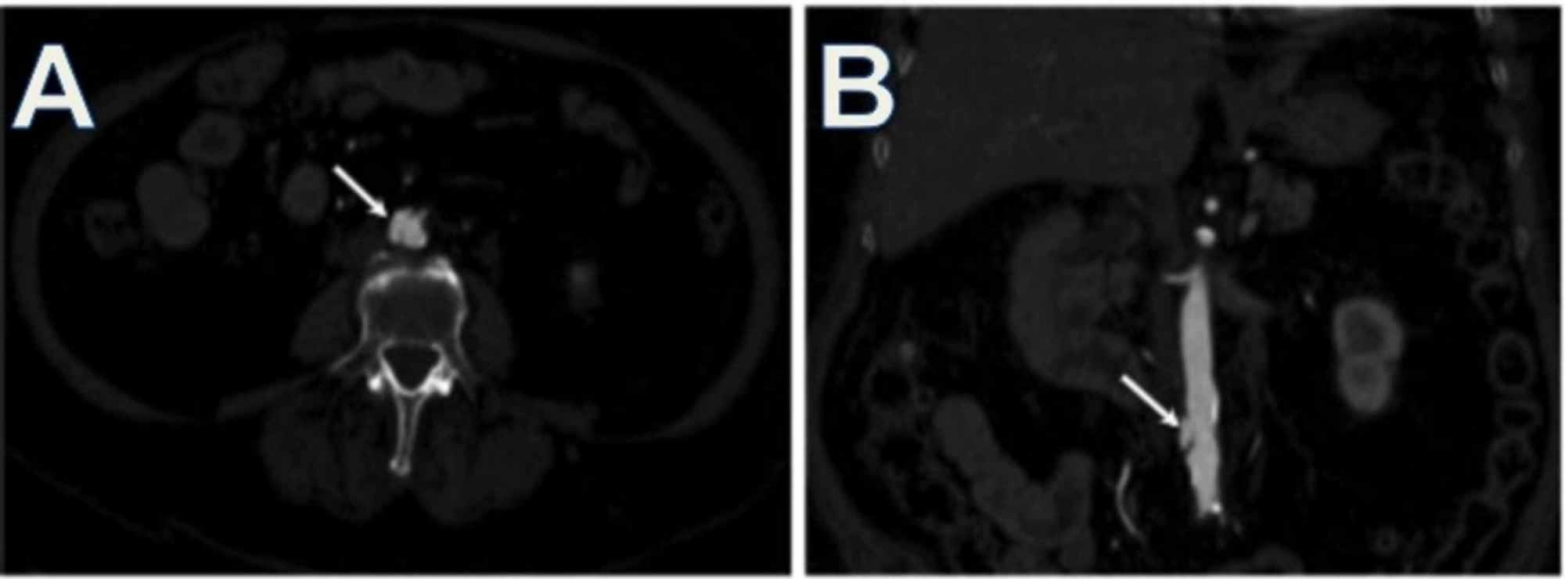
Penetrating Atherosclerotic Ulcer Axial image (A) and coronal reformat (B) of the contrast-enhanced CT demonstrate an atheromatous plaque in the abdominal aorta extending beyond the intima into the aortic media. PAU will increase the patient’s risk of intramural hemorrhage, pseudoaneurysm, or dissection formation.

In extensive atherosclerosis, high-density hematoma surrounding the ulceration and variable size IMHs can be detected in non-contrast enhanced images. In contrast-enhanced images, ulceration of the atherosclerotic plaque can be seen and would show protrusion beyond the intimal level into the medial layer of the aortic wall, together with a focal outpouching of the outer aortic contour. The protrusion and focal contour change may differentiate the PAU from the common atheromatous ulcer (Figure 5) [36,44].

Invasive treatments such as surgery and stent-grafting are required in acute and symptomatic cases, but course observation, including periodical evaluation using imaging techniques is recommended in asymptomatic or chronic cases [41,45].

​​​Aortic aneurysm and rupture

Aortic aneurysmal enlargement is defined as a permanent dilatation to at least 150% of the normal size. According to their contents, aneurysms are divided into two: true and false. “True” aneurysms include all the layers of the aortic wall whereas “false” aneurysms are contained ruptures and usually comprise just the adventitia, surrounded by fibrosis and hematoma. Localized aneurysms are usually divided into two: “saccular” and “fusiform,” with fusiform defined by more diffuse dilatation [2,5,46]. Most of the aneurysms involve the aortic isthmus; aneurysm rupture occurs when the mechanical stress on the wall exceeds the strength of the wall tissue. The main events of the rupture of the aneurysm include the formation of an intramural hematoma and hemorrhagic leak into the mediastinum through the aortic leak, with the progressive invasion of the pleural cavity, and pericardium. Sometimes, the hematoma may separate the parietal pleura from the endothoracic fascia, leading to an extrapleural hematoma [47-49].

Hyperdense thickening of the aortic wall that represents blood collection between partially disrupted wall layers and mediastinal hematoma is usually detected on MDCT angiography images. This mediastinal hematoma may extend from the site of the aortic lesion into the periaortic mediastinal fat. Pleural and rarely pericardial effusion can also be detected on MDCT images. MDCT angiography also may be helpful to depict signs of impending hypovolemic shock via presenting reduction in the caliber of central vessels and excessive contrast enhancement of the aorta relative to the injection parameters [9,11,47-48]

## Conclusions

Acute aortic syndromes are important emergency conditions of the aorta and can often lead to the patient’s death. Early diagnosis and treatment are essential for improving prognosis. MDCT allows the imaging of the entire aorta with rapid acquisition and data reconstruction to provide a prompt and accurate diagnosis, including all of the AAS types and helps identify relevant complications that may have an impact on surgical planning or management.
